# A characterization of trauma laparotomies in a scandinavian setting: an observational study

**DOI:** 10.1186/s13049-022-01030-4

**Published:** 2022-07-08

**Authors:** Jakob Mejdahl Bentin, Emma Possfelt-Møller, Peter Svenningsen, Søren Steemann Rudolph, Martin Sillesen

**Affiliations:** 1 Department of Anesthesia, Center of Head and Orthopedics, Rigshospitalet, Copenhagen, Denmark; 2 Department of Organ Surgery and Transplantation, Copenhagen University Hospital, Rigshospitalet, Denmark; 3 Department of Surgical Gastroenterology, North Zealand Hospital, Hillerød, Denmark; 4 Institute of Clinical Medicine, University of Copenhagen, Blegdamsvej 3b, 2200 Copenhagen N, Denmark

**Keywords:** Trauma, Abdominal injury, Laparotomy, Scandinavia, Mortality, Retrospective cohort

## Abstract

**Background:**

Despite treatment advances, trauma laparotomy continuous to be associated with significant morbidity and mortality. Most of the literature originates from high volume centers, whereas patient characteristics and outcomes in a Scandinavian setting is not well described. The objective of this study is to characterize treatments and outcomes of patients undergoing trauma laparotomy in a Scandinavian setting and compare this to international reports.

**Methods:**

A retrospective study was performed in the Copenhagen University Hospital, Rigshospitalet (CUHR). All patients undergoing a trauma laparotomy within the first 24 h of admission between January 1st 2019 and December 31st 2020 were included. Collected data included demographics, trauma mechanism, injuries, procedures performed and outcomes.

**Results:**

A total of 1713 trauma patients were admitted to CUHR of which 98 patients underwent trauma laparotomy. Penetrating trauma accounted for 16.6% of the trauma population and 66.3% of trauma laparotomies. Median time to surgery after arrival at the trauma center (TC) was 12 min for surgeries performed in the Emergency Department (ED) and 103 min for surgeries performed in the operating room (OR). A total of 14.3% of the procedures were performed in the ED. A damage control strategy (DCS) approach was chosen in 18.4% of cases. Our rate of negative laparotomies was 17.3%. We found a mortality rate of 8.2%. The total median length of stay was 6.1 days.

**Conclusion:**

The overall rates, findings, and outcomes of trauma laparotomies in this Danish cohort is comparable to reports from similar Western European trauma systems.

**Supplementary Information:**

The online version contains supplementary material available at 10.1186/s13049-022-01030-4.

## Background

Major trauma remains a significant cause of morbidity and mortality. The World Health Organization (WHO) estimates that trauma is the direct cause of 9% of the mortality worldwide [1]. Abdominal trauma accounts for an estimated 10% of the burden of injury in Europe [2]. Although the distinction between blunt and penetrating trauma is important, both frequently require surgical exploration in the form of a trauma laparotomy.

Historically, the mortality rate of patients undergoing trauma laparotomy was 40% [3]. However, more recent literature shows significantly lower mortality rates between 7 and 21% [4–10]. These favorable outcomes may in part be attributed to recent advances in concepts such as Damage Control Surgery (DCS) and Damage Control Resuscitation (DCR), which is now an integral part of the treatment strategy for critically injured trauma patients to reduce the risk of morbidity and mortality [11–17].

While these concepts are now widely adopted, differences in protocol adherence as well as the underlying patient demographics, including the percentage of penetrating versus blunt trauma patients, may impact on outcomes [18]. Furthermore, comparisons between centers as well as assessments of the driving factors of adverse outcomes from aggregated trauma databases is difficult, a fact that has been demonstrated when European trauma systems are compared [19].

Understanding of the underlying demographics and adherence to treatment protocols are thus critical when analyzing trauma outcomes. While these factors are well documented for trauma systems in the United Kingdom (UK) and United States (US) [20, 21], less is known about Scandinavian trauma systems.

The objective of this study is to characterize treatments and outcomes of patients undergoing trauma laparotomy in a level 1 trauma center in Denmark. We hypothesize that outcomes and adherence to protocols are comparable with internationally published reports from comparable trauma systems.

## Methods

This is a retrospective quality assurance study of trauma patients admitted to Copenhagen University Hospital, Rigshospitalet Trauma Centre (CUHR TC) between January 2019 and December 2020. The study was conducted and reported in line with STROBE guidelines.

Access to patient data for the purpose of quality assurance was approved by the local Ethics Board (ID: PID 3714). The CUHR Trauma Centre meets the Level 1 Trauma Centre standards according to the American College of Surgeons. It serves as a regional trauma center for the eastern part of Denmark with a population of 2.6 million people (46% of the Danish population).

### Patient selection

We included all patients admitted to the CUHR Trauma Center, both primary and secondary admissions, undergoing a trauma laparotomy (including both in the operating room and trauma bay) within the first 24 h of admission between January 1st 2019 and December 31st 2020. Data were obtained from the local trauma registry and the EPIC electronic health record system (Verona, WI, USA) used within the Capital Region of Denmark.

### Study variables

Extracted data included demographics and prehospital data: sex, age, trauma mechanism (blunt/penetrating), Injury Severity Score (ISS) [22], American Society of Anesthesiologists (ASA) score [23], Glasgow Coma Scale (GCS) [24], Abbreviated Injury Scale (AIS) [25], time from injury to arrival at trauma care unit and trauma call duration.

In hospital data included systolic blood pressure at arrival, blood lactate at arrival, radiological procedures performed, blood transfusions, indication for surgery, place of surgery (trauma bay versus designated operating room), time from arrival in the Trauma Center to surgery start, duration of surgery, DCS approach (including indication for DCS), charge of most senior abdominal surgeon, findings during laparotomy, procedures performed as well as hemodynamic status during surgery.

Post laparotomy data included mortality, total number of operations needed in any region, intensive care unit (ICU) length of stay (LOS), hospital LOS, discharge destination to (home, local hospital, nursing home).

### Data and statistical analysis

Continuous data are presented as medians (interquartile range (IQR)). Dichotomous and categorial data are presented as percentages. All statistical analyses were performed using Microsoft Excel (Microsoft, Redmond, WA, USA. Version 16.0.14026.20304).

### Data integrity and collection

All data were collected by JMB and verified by EPM and SSR. All data were registered in the Research Electronic Data CAPture (RedCAP) system (Vanderbilt University, Nashville, TN, USA).

### Missing data

Missing data were considered missing at random. Supplementary table 1 provides an overview of the percentage of missing data points for each registered variable.

## Results

During the 2-year study period, 1713 trauma patients were admitted to CUHR. These were either directly admitted (n = 87, 88.8%) or transferred from one of 15 referral hospitals. Of these, 571 (33.3%) had an ISS > 15. Blunt trauma accounted for 83.4%, whereas penetrating injuries accounted for 16.6%. We identified 98 patients eligible for study inclusion after having screened the total number of trauma patients in the study period (1713) and excluded the 1615 who did not have a trauma laparotomy performed. Demographic and injury characteristics data are presented in Table 1.

**Table 1:** Patient demographics and injury characteristics for the 98 trauma laparotomy patients

Parameter	Number of patients	Distribution
**Sex**		
Male	79	80.6%
Female	19	19.4%
Age		31 (25) years
**Trauma mechanism**		
Penetrating	65	66.3%
Blunt	33	33.7%
**Damage mechanism**		
Blows and shocks (incl. fall from height and same level)	9	9.2%
Gunshot	6	6.1%
Stab, cut, bite	59	60.2%
Traffic accidents	24	24.5%
Other	2	2.0%
**Type of admission**		
Primary	87	88.8%
Secondary	11	11.2%
ISS		13 (17)
GCS		15 (2)
**AIS regions**		
AIS head	NA	0 (0)
AIS face	NA	0 (0)
AIS chest	NA	1 (3)
AIS abdomen	NA	3 (1)
AIS extremities	NA	0 (1)
AIS external	NA	0 (1)
Systolic blood pressure at arrival	NA	113 (40) mmHg
Lactate at arrival	NA	2.9 (4.1)
ASA classification (pre-injury)	NA	1 (1)
**Radiology before surgery**		
eFAST performed	69	70.4%
Positive	25	36.2%
CT	67	68.4%
Selective trauma scan	19	28.4%
Full trauma scan	47	70.1%
Time to CT after arrival		20 (13) min
**Transfusion**		
Total volume of transfused blood product		2000 (3750) ml
Patients received PRBC	34	34.7%
Patients received FFP	37	37.8%
Patients received PLT	29	29.6%
Time from injury to arrival at trauma care unit	54 (43) min	54 (43) min
Trauma call duration	58 (57) min	58 (57) min

Numbers indicate percentages or medians (interquartile range) where appropriate

*NA* not applicable, *ISS* injury severity score, *GCS* glasgow coma scale, *AIS* abbreviated injury scale, *ASA* American Society of Anesthesiologists score, *eFAST* extended focused assessment with sonography for trauma, *CT* computed tomography scan, *PRBC* packed red blood cells, *FFP* fresh frozen plasma, *PLT* platelets

In the study population, penetrating trauma was the predominant mechanism of injury (66.3%) (Table 1). Stabbing was the most common mechanism of injury (n = 59, 60.2%), while gunshot wounds only accounted for a minor part of the cases (n = 6, 6.1%). Road traffic collisions was the predominant mechanism in blunt trauma (n = 24, 24.5%). Motor vehicle accidents being most common, followed by pedestrians, cyclists, and motorcycles. The median ISS score within the study population was 13 (16), with 43 (43.9%) having an ISS > 15.

Table 2 provides information on identified injuries and treatments deployed.

**Table 2:** Identified injury patterns and patient treatment

Parameter	Number of patients	Distribution
**Place of surgery**		
Trauma bay	14	14.3%
Operating room	84	85.7%
**Time to surgery after arrival**		
Trauma center	NA	12 (20) min
Operating room	NA	103 (88) min
**Duration of surgery**		
Trauma center	NA	20 (53) min
Operating room	NA	88 (68) min
**Charge of senior surgeon**		
Resident	23	23.5%
Attending	75	76.5%
**Indication for surgery**		
Cardiac arrest	7	4.3%
Haemodynamic instability/shock	27	16.8%
Penetrating trauma	64	39.8%
Free air	21	13.0%
Free fluid	38	23.6%
Peritoneal reaction	4	2.5%
**Damage control surgery**		
Yes	18	18.4%
No	80	81.6%
**Indication for damage control surgery**		
Haemodynamic instability/shock	7	38.9%
Planned second look	8	44.4%
Severe metabolic derangement and/or hypothermia	3	16.7%
**Initiated as laparoscopy**		
Yes	12	12.2%
No	86	87.8%
Blood loss	NA	365 (1360) ml
**Injuries**		
Negative laparotomy	17	17.3%
Stomach, anterior and posterior surface	14	14.3%
Spleen	12	12.2%
Duodenum	4	4.1%
Small intestine	19	19.4%
Mesentery of small intestine	24	24.5%
Colon	22	22.4%
Mesentery of colon	14	14.3%
Liver/gall bladder	26	26.5%
Pancreas	5	5.1%
Diaphragm	9	9.2%
Retroperitoneum zone I	15	15.3%
Hematoma	14	93.3%
Aorta	2	13.3%
Vena cava	3	20.0%
Retroperitoneum zone II	22	22.4%
Right	12	54.5%
Left	10	45.5%
Hematoma	16	72.7%
Kidney	11	50.0%
Adrenal gland	2	9.1%
Renal vein	1	4.5%
Renal artery	2	9.1%
Ureter	0	0%
Retroperitoneum zone III	6	6.1%
Hematoma	5	83.3%
Iliaca/ureteres	1	16.7%
Urinary bladder	0	0%
Fecal contamination	2	2.0%
Intraperitoneal lesion of rectum	1	1.0%
Extraperitoneal lesion of rectum	0	0%
**Procedures**		
Abdominal packing	16	16.3%
Vacuum Assisted Closure	16	16.3%
Intestinal resection	15	15.3%
Splenectomy	8	8.2%
Hemostatic procedure on the spleen	3	3.1%
Hemostatic procedure on the liver	14	14.3%
Suture of intestine/stomach	31	31.6%
Suture of blood vessel	10	10.2%
Shunt of blood vessel	0	0%
Nefrectomy	1	1.0%
Suture of urinary bladder	0	0%
Enterostomy	1	1.0%
Suture of diaphragm	7	7.1%
Surgical drain	29	29.6%
Endoscopy	1	1.0%
Anastomosis	8	8.2%
Hemostatic procedure on mesentery	4	4.1%
Suture of mesentery	11	11.2%
Cholecystectomy	1	1.0%
**Haemodynamic**		
Cardiac arrest during procedure	7	7.1%
Systolic blood pressure at "start of surgery"	NA	90 (20) mmHg
Lowest systolic blood pressure during surgery	NA	75 (16) mmHg
Mean arterial pressure at "start of surgery"	NA	62 (20) mmHg
Lowest mean arterial pressure during surgery	NA	50 (9) mmHg
Temperature at "start of surgery"	NA	36.1 (1.4) celcius
Lowest temperature during surgery	NA	35.8 (1.5) celcius
Lowest lactate during surgery	NA	2.2 (1.5)
Highest lactate during surgery	NA	2.5 (1.9)

*NA* not applicable

Frequent injuries included damage to the mesentery of small intestine (n = 24, 24.5%), colon (n = 22, 22.4%), liver/gall bladder (n = 26, 26.5%) and retroperitoneum zone II (n = 22, 22.4%). Damage to duodenum, pancreas, diaphragm, retroperitoneum zone III and fecal contamination were all rare (below 10%). In 17 (17.3%) cases no injuries were identified during surgery. On further investigation of the negative laparotomy cases, 4 (23% of negative laparotomies) were crash laparotomies on exsanguinating patients concurrent with thoracotomies where intraabdominal injury was suspected but not found. The remainder (n = 13, 77% of negative laparotomies) were due to institutional protocols mandating laparotomy following evidence of peritoneal penetration for stab wounds by either diagnostic laparoscopy, clinical examination, or CT findings.

The most frequent interventions were suture of intestine/stomach (n = 31, 31.6%) and intestinal resection (n = 15, 15.3%) followed by hemostatic procedure on the liver (n = 14, 14.3%). Primary anastomosis was made in 8 (8.2%) patients, and primary enterostomy in 1 (1.0%) patient. Abdominal packing was only needed in 16 (16.3%) cases, vacuum assisted closure (VAC) was chosen for 16 (16.3%) patients, and of these 10 patients received both abdominal packing and VAC.

The median amount of transfused blood product was 2000 (3750) ml. 34 patients (34.7%) received Packed Red Blood Cells (PRBC), 37 patients (37.8%) Fresh Frozen Plasma (FFP) and 29 patients (29.6%) Platelets (PLT). Massive transfusion protocol (MTP) was activated in 34 of these patients. Our institution adheres to an MTP protocol dictating 1:1:1 transfusion ratios until viscoelastic guided transfusions are available.

Table 3 provides an overview of postoperative and outcome data. We found a mortality rate of 8.2% (n = 8) with 6 being in the trauma bay and 2 in addition during hospitalization. The median time to death after arrival to the hospital was 46 (135) minutes. The most frequent cause of death was hemorrhage (n = 4, 50%). For patients presenting in hemorrhagic shock, defined as a first measured systolic blood pressure < 90 mmHg in the trauma bay, the overall mortality rose to 31.8%.

**Table 3:** Postoperative and outcome data

Parameter	Number of patients	Distribution
**Mortality**		
Death in ED	6	6.1%
Death following ED discharge	2	2.0%
Discharged alive	90	91.8%
Time to death after arrival	NA	46 (135) min
Cause of death		
Traumatic brain damage	1	12.5%
Bleeding	4	50.0%
Other	1	12.5%
Unknown	2	25.0%
**Destination after trauma care unit**		
Intensive care unit	53	59.6%
Ward	36	40.4%
**Length of stay**		
Intensive care unit	NA	2.0 (6.1) days
Hospital	NA	6.1 (11.1) days
**Number of surgical interventions during primary admission in any region**		
1	51	59.3%
2	14	16.3%
3	11	12.8%
> 3	10	11.6%
**Discharged to**		
Own home	46	54.8%
Another hospital	17	20.2%
Another intensive care unit	3	3.6%
Psychiatric department	14	16.7%
Rehabilitation, Spinal Cord Injury	1	1.2%
Rehabilitation, Traumatic Brain Injury	1	1.2%
Rehabilitation, other	2	2.4%

*NA* not applicable, *ED* emergency department

Most patients were admitted to the ICU after the trauma resuscitation and surgery (n = 53, 59.6%), where the median length of stay was 2.0 (6.1) days. The total median length of stay was 6.1 (11.1) days.

## Discussion

Penetrating injury is infrequent in most western European countries, whereas higher rates are seen in the US and South Africa [26, 27]. In the Northern European countries, penetrating trauma is seen in 5–14% [28–32], and 37–58% [29, 33, 34] for patients undergoing trauma laparotomy, with gunshots accounting for 5–36% [28, 35–38]. In our study, penetrating trauma accounted for 16,6% of the trauma population and 66.3% for patients undergoing laparotomy. This is higher, when compared to European and Scandinavian countries, but is comparable to other countries outside Europe [4, 6, 7, 10, 34, 39–43].

Compared to other studies, our population of predominantly younger males is comparable [4, 6, 7, 10, 34, 39, 40, 43–46], with ISS being slightly lower than other published cohorts [4, 6, 7, 10, 34, 39, 40, 43, 45, 46]. In terms of injuries, we identified an overrepresentation of bowel-, liver- and stomach injuries, and an overall mortality rate of 8.2%. Internationally, trauma laparotomy mortality rates have been reported between 6 and 21% [4, 6, 7, 10, 34, 39, 43, 45, 46], but care should be taken when comparing these rates, as differences in patient demographics and injury modalities likely impact on results. The reported LOS at the ICU of 2 days and total in-hospital LOS of 6.1 days is in line with previously reported findings [4, 6, 7, 34, 39, 43, 45].

Of interest, mortality rates rose to 31.8% for patients presenting to our hospital in hemorrhagic shock. These rates are lower than rates reported from both US and UK centers, ranging consistently between 46–47% [10, 47], but higher than rates from other US centers reporting a mortality rate of 18%[48]. Although it is tempting to conclude on the lower mortality rates for patients presenting in hemorrhagic shock identified here compared to US and UK reported rates, care should be taken as multiple both treatment and patient demographic factors could have influenced findings. Furthermore, it is important to underline that this study presents data from a limited group of patients, and that variations in mortality rates across the study period could affect the presented data disproportionally.

Collectively, this Danish trauma laparotomy cohort is thus comparable to other European centers in terms of demographics and overall outcomes but seem to differ somewhat in terms of injury modalities, with penetrating trauma accounting for a higher percentage of laparotomy indications. Rather than owing to differences in actual injury modalities, this may also be partly attributed to other factors. One factor is that a greater availability of interventional radiology provides the possibility to omit the need for laparotomy in for instance many blunt liver or splenic injury trauma patients. In this cohort, no laparotomies were preceded by interventional radiology. Another factor is differences in adherence to protocols dictating the need for surgical exploration in patients with suspected abdominal fascia penetration but without associated signs of injuries (e.g., peritonitis or hypotension). This again is underlined by the fact that our negative laparotomy rates (17.3%) are much higher than reported rates of 3.9% from international high-volume centers[49], thus indicating an increased use of this approach in our cohort. Other reports have, however, indicated a wide range in negative laparotomy incidences ranging from 6 to 36% [7, 41, 42, 45, 49–55]. A further analysis of the negative laparotomy patients revealed that 77% of these were operated solely due to evidence of peritoneal penetration with signs of associated injury, a protocol that remains debatable and not supported by neither the Eastern Association of Trauma (EAST), Western Trauma Association (WTA) or the World Society of Emergency Surgery guidelines [56–58].

In terms of the treatment flow, we identified a median time to trauma laparotomy in the ED and OR of 12 (20) minutes and 103 (88) minutes respectively. While no definite limit for time to emergency laparotomy exists, the American College of Surgeons Committee on Trauma (ACS-COT) has previously recommended an audit filter of 2 h [59], with other studies reporting median times from ED admission to surgical start of 24–56 min [10, 40, 44]. The observed delays to OR in this cohort is thus within acceptable limits but could be influenced by the fact that our trauma team also oversee other non-trauma clinical duties when on call, as well as the fact that we do not have a dedicated trauma operating room on 24/7 standby. Of interest, care systems and organizational changes, e.g., a specialist trauma surgeon, have been associated with significantly improved outcomes [21, 43, 60–62].

A DCS approach was used in 18% of cases, which is low compared to other studies [7, 10, 34, 39]. While this could potentially reflect an underuse of the DCS approach in otherwise eligible patients, it should be interpreted in the light of the above-mentioned findings of high negative laparotomy rates. In patients where the DCS approach was deployed, duration of surgery was 20 (53) minutes for procedures done in the ED, and 88 (68) minutes for procedures done in the OR, which is reasonably in line with published guidelines and previous reports of average operation times of 62 min [7]. Overall, 14% of patients were operated in the ED, with other reports indicating rates of 22 to 51% [40, 63]. Again, rather than reflecting upon an underuse of ED laparotomies, these rates should be interpreted in the light of the high negative laparotomy rates, thus indicating a difference in the injury severity of patients.

Potentially both negative laparotomy rates as well as overall outcomes could be contingent upon the surgical experience of the care provider team. ACS-COT has set a standard of 35 cases per year per surgeon [64], with studies identifying an association between the average surgeon’s volume of seriously injured patients and mortality for all patients [65], as well as associations between trauma center volume and mortality [65–70]. In this cohort, the charge of the most senior surgeon was in 23% of the cases a senior resident, with an estimated annual caseload of 6 trauma laparotomies based on-call frequency and the findings identified here. With a previously reported association between trauma center volume and laparotomy outcomes [71], an outcome benefit could potentially be realized by increasing the caseload for the trauma center as well as individual surgeon through trauma center referral criteria as well as the formation of a dedicated trauma laparotomy team. It is, however, important to underline that the current caseload does not support the notion of forming a dedicated trauma surgery team, and that increased trauma treatment centralization would be required for this to become a realistic option.

### Limitations

The interpretation is limited by the retrospective nature of the data. This makes the study dependent on data quality and granularity, factors that would be optimal if approached through a prospective study. Furthermore, data are derived from a single center, thus no conclusions can be drawn on the overall trauma system in Denmark. Additionally, the study setup does not allow for a direct statistical comparison with other centers, and multiple confounding factors could thus affect results when direct comparisons with reported data from other centers is done. To this end, extended scoring systems in addition to the ISS allowing for center comparisons such as the Trauma Injury Severity Score (TRISS) would have been of value. Unfortunately, available data did not allow for the calculation of this score. Finally, the limited number of trauma laparotomies coupled with the high rate of negative laparotomies is a weakness of the study, and results should be viewed considering this.

## Conclusions

The overall rates, findings, and outcomes of trauma laparotomies in this Danish cohort, is comparable to reports from similar Western European trauma systems but differ from reports from centers where rates of penetrating traumas are higher. Furthermore, we identified potential areas of quality improvement, where future focus should be directed.

## Supplementary Information

**Additional file 1. Supplemantary table 1:** Overview of missing data for relevant data points.

## Data Availability

The datasets used and/or analysed during the current study are available from the corresponding author on reasonable request.

## Abbreviations

CUHR: Copenhagen University Hospital, Rigshospitalet
ISS: Injury Severity Score
ASA: American Society of Anesthesiologists Score
GCS: Glasgow Coma Scale
AIS: Abbreviated Injury Scale
DCS: Damage Control Surgery
VAC: Vacuum assisted closure
FFP: Fresh Frozen Plasma
PRBC: Packed Red Blood Cells
PLT: Platelets
OR: Operating Room
ED: Emergency Department
ACS-COT: American College of Surgeons, Committee on Trauma
