# Supplementary material for: A characterization of trauma laparotomies in a scandinavian setting: an observational study
Source: Scand J Trauma Resusc Emerg Med. 2022 Jul 8;30:43. doi: 10.1186/s13049-022-01030-4 (PMC9264678; doi:10.1186/s13049-022-01030-4)
Supplement: Supplementary file 1 — Additional file 1. Supplemantary table 1: Overview of missing data for relevant data points. [file 13049_2022_1030_MOESM1_ESM.docx]

**Supplementary table 1: Missing data**

| **Parameter** | |  | **Missing (n, %)** |
| --- | --- | --- | --- |
| **Table 1** |  |  |  |
| Sex |  |  | 0 |
| Age |  |  | 0 |
| Trauma mechanism | |  | 0 |
| Damage mechanism | | | 0 |
| ISS |  |  | 0 |
| GCS |  |  | 6, 6.1% |
| AIS regions | |  | 0 |
| Systolic blood pressure at arrival | | | 4, 4.1% |
| Lactate at arrival | |  | 17, 17.3% |
| ASA classification | |  | 0 |
| Radiology before surgery | | | 0 |
|  | eFAST |  | 2, 2.9% |
|  | CT |  | 1, 1.5% |
| Transfusion | |  | 7, 7.1% |
| Time from injury to arrival at trauma care unit | | | 36, 36.7% |
| Trauma call duration | | | 0 |
|  |  |  |  |
| **Table 2** |  |  |  |
| Place of surgery | |  | 0 |
| Time to surgery after arrival | | |  |
|  | Traume center | | 3, 21.4% |
|  | Operating room | | 0 |
| Duration of surgery | |  | 0 |
| Charge of senior surgeon | | | 0 |
| Indication for surgery | | | 0 |
| Damage control surgery | | | 0 |
| Indication for damage control surgery | | | 0 |
| Initiated as laparoscopy | | | 0 |
| Blood loss | |  | 11, 11.2% |
| Injuries |  |  | 0 |
| Procedures | |  | 0 |
| Haemodynamic | |  |  |
|  | Cardiac arrest during procedure | | 0 |
|  | Systolic blood pressure at "start of surgery" | | 4, 4.1% |
|  | Lowest systolic blood pressure during surgery | | 4, 4.1% |
|  | Mean arterial pressure at "start of surgery" | | 4, 4.1% |
|  | Lowest mean arterial pressure during surgery | | 4, 4.1% |
|  | Temperature at "start of surgery" | | 26, 26.5% |
|  | Lowest temperature during surgery | | 19, 19.4% |
|  | Lowest lactate during surgery | | 26, 26.5% |
|  | Highest lactate during surgery | | 26, 26.5% |
|  |  |  |  |
| **Table 3** |  |  |  |
| Mortality |  |  | 0 |
| Destination after trauma care unit | | | 3, 3.3% |
| Length of stay | |  | 2, 2.2% |
| Number of surgical interventions during primary admission | | | 12, 12.2% |
| Discharged to | |  | 6, 6.7% |

*ISS: Injury Severity Score; GCS: Glasgow Coma Scale; AIS: Abbreviated Injury Scale; ASA: American Society of Anesthesiologists score; eFAST: Extended Focused Assessment with Sonography for Trauma; CT: Computed tomography scan.*
